# A Review on Biomaterials for Orthopaedic Surgery and Traumatology: From Past to Present

**DOI:** 10.3390/ma15103622

**Published:** 2022-05-18

**Authors:** Grzegorz Szczęsny, Mateusz Kopec, Denis J. Politis, Zbigniew L. Kowalewski, Adam Łazarski, Tomasz Szolc

**Affiliations:** 1 Department of Orthopaedic Surgery and Traumatology, Medical University, 4 Lindleya Str., 02-005 Warsaw, Poland; grzegorz.szczesny@wum.edu.pl (G.S.); adam.lazarski@wum.edu.pl (A.Ł.); 2 Institute of Fundamental Technological Research, Polish Academy of Sciences, 5B Pawińskiego Str., 02-106 Warsaw, Poland; zkowalew@ippt.pan.pl (Z.L.K.); tszolc@ippt.pan.pl (T.S.); 3 Department of Mechanical and Manufacturing Engineering, University of Cyprus, Nicosia 20537, Cyprus; politis.denis@ucy.ac.cy

**Keywords:** orthopaedic surgical procedures, biomaterials, implants, biocompatible materials, alloys, ceramic, polyethylene

## Abstract

The principal features essential for the success of an orthopaedic implant are its shape, dimensional accuracy, and adequate mechanical properties. Unlike other manufactured products, chemical stability and toxicity are of increased importance due to the need for biocompatibility over an implants life which could span several years. Thus, the combination of mechanical and biological properties determines the clinical usefulness of biomaterials in orthopaedic and musculoskeletal trauma surgery. Materials commonly used for these applications include stainless steel, cobalt-chromium and titanium alloys, ceramics, polyethylene, and poly(methyl methacrylate) (PMMA) bone cement. This study reviews the properties of commonly used materials and the advantages and disadvantages of each, with special emphasis on the sensitivity, toxicity, irritancy, and possible mutagenic and teratogenic capabilities. In addition, the production and final finishing processes of implants are discussed. Finally, potential directions for future implant development are discussed, with an emphasis on developing advanced personalised implants, according to a patient’s stature and physical requirements.

## 1. Introduction

Anaesthesia and asepsis, together with improvements in industrial technology that occurred in the second half of the 19th century, rapidly accelerated a progress in the field of surgery. Such progress promoted the introduction of new techniques and methods, that enabled chirurgical interventions into practically every region of the body. In recent decades, skeletal procedures including implants have dramatically improved quality of life by implementing supporting structures to withstand mechanical loads for the support of bone fractures or replacing irreversibly damaged bones entirely. As a direct consequence of these procedures, specialised implants and suitable materials have been developed to address these needs. Unfortunately, the materials used in early interventions were characterised by inadequate durability, low biocompatibility, and limited availability [1]. The earliest attempts to use materials including wood, leather, cotton, silk, coral, animal bones and ivory, bitumen, glass, Pyrex, Bakelite, and Formica laminate, as well as ceramic, provided unsatisfactory results [2]. Metals were regarded as much more promising due to their considerably higher toughness. Copper and its alloys, although of low cost and possessing desirable bactericidal properties, were not durable enough to carry the body weight. Additionally, in contact with the biological environment, these materials produce highly irritant, and even toxic, salts [3]. Other materials, such as gold, silver, platinum, and ruthenium, and their salts, were found irritant and toxic as well; however, their irritancy and toxicity were far less prominent, but still sufficient to control microbial growth [3,4]. Thus, these metals are scarcely found in bone surgery, even though some have been adopted for dentistry and soft tissue reconstructive surgery. Iron and its alloys were among the most promising materials due to their low cost, practically unlimited availability, and sufficient mechanical properties. However, the high rate of unwanted side effects (tendency to corrosion, low biocompatibility, and subsequent tissue irritation) limited their application despite their excellent manufacturability for complex shapes such as limb prosthetics [3]. The consequences of metals insertion (usually iron, copper, and bronze) into viable tissues has been known for centuries. High susceptibility to corrosion, tissue irritation, and suppuration limited their medical applications [4]. The first use of surgical implants made with steel was attributed to Sherman (1912) [5]. Sherman vanadium steel, despite exhibiting a relatively high hardness, was characterised with inadequate corrosion resistance in a biological environment jeopardizing vanadium intoxication. Subsequent progress in metallurgy in the 19th and 20th centuries introduced several alloys with desirable mechanical and biological characteristics. Steel and its stainless alloys (Brearley in UK and Krupp in Germany, 1913) developed at the beginning of 20th century are still used with minor chemical modifications even to the present day. Based on steels developed during this period, the 18/8 stainless steel (Hatfield, UK, 1924), a direct precursor to 316 L steel, was widely known for its medical applications as a “surgical” steel. Stainless steel was also used for the production of the first widely used orthopaedic implants and devices, including Lambotte external fixators, Kirschner wires, Rush nails, bone plates and screws, Kuntcher intramedullary nails, Austin Moore hip prostheses, and many others. Improvements in ore acquisition technology, smelting, and purification have enabled high quantities of metals and new alloys to be developed, some of which have found biomedical applications. The most extensively used modern alloys include cobalt–chromium (with or without molybdenum) alloys and titanium alloys. On the other hand, ceramics, as non-corrosive materials with excellent biocompatibility, low degradability, high melting temperature, and improved mechanical properties with limited plasticity in comparison to metal-based biomaterials have been successfully used in dentistry, orthopaedics, calcified tissues, implants, coatings, medical sensors, and many other applications [6]. Along with ceramics, polymers have been used as biomaterials in orthopaedic surgery for decades. Biocompatible polymers have been used successfully in total joint replacements, for soft tissue reconstruction, joint fusion, and as fracture fixation devices [7].

Continuous development of both medicine and materials for orthopaedic surgery and traumatology require the knowledge of their mechanical and microstructural properties. Although some fundamental information is known to clinicians, the understanding of material behaviour is still limited. Therefore, the main aim of this paper is to discuss the relations between material properties and implant performance. Biomaterials currently used for orthopaedic and traumatology treatments were analysed in terms of their chemical composition, biocompatibility, mechanical properties, and manufacturing technologies.

## 2. Materials and Methods

The literature review conducted as part of this study involved the detailed investigation of conventional biomaterials for orthopaedic surgery and traumatology with particular emphasis on their historical aspects. It was conducted in the PubMed and Web of Science databases with the following keywords used in various combinations: “biomaterials, implants, orthopaedic surgical procedures, biocompatible materials, including steel, polymers, alloys, ceramic, polyethylene, as well as their mechanical properties, applications, implantology and surface modification”. Only research papers published in English language were included. A number of 380 articles were found through the electronic databases. All the collected studies were considered by independent reviewers who assess the eligibility of studies by screening the title, the abstract and the summary of each paper using the pre-specified inclusion and exclusion criteria. Subsequently, the studies not fitting the inclusion criteria were excluded. A total number of 110 articles being found as relevant for the purposes of this review. The recent trends in biomaterials were not discussed as the main aim of this review was to provide a historical perspective through a review of documentation on conventional biomaterials across the last century. This work will principally focus on the most relevant research published between 1950 and 2020.

## 3. Biomaterials for Orthopaedic Surgery and Traumatology

### 3.1. Metallic Biomaterials

#### 3.1.1. Steel

Stainless “surgical” steel remains one of the most frequent applied alloys to manufacture surgical implants and instruments. These alloys serve for the fabrication of at least half of all orthopaedic implants used in the USA [8], although they are gradually being displaced from the market by other alloys, notably CoCrMo and titanium alloys.

Stainless steel possesses several desirable properties. This material is durable, ductile, and, thus, relatively simple to process. It is also non-toxic and biocompatible, as it does not evoke an adverse reaction from adjacent tissues. Technology of its production (smelting, casting, and forging) and processing (cold-hammering, tempering, machining, and threading) is well known and relatively low cost. The final products made of steel are available in practically unlimited quantities for an acceptable price. 

Nowadays, an austenitic 316 L steel is mainly used for implants due to its high corrosion resistance. It consists of reduced carbon (below 0.03%) and increased chromium (16–18%) and nickel (10–14%) content, with the addition of molybdenum (2–3%), manganese (ca 2%), and small additives of sulphur, silicon, phosphorus, and nitrogen [9]. Corrosion resistance results from a thin Cr_2_O_3_ layer, that passivates on the outer layer. Such layers protect the human organism as the implant does not interfere with metabolic processes, that occur in the body. It should be mentioned, however, that there is the possibility for exceedingly high chromium and nickel content to lead to several unwanted side effects, as both may irritate tissues and lead to immune reactions. This has been demonstrated in the literature where up to 20% of the population of industrialised countries demonstrate sensitivity to the chromium and nickel [9]. Chromium and nickel may be carcinogenic, and their high concentration may even be toxic [10,11] and may promote infections caused by nickel-dependent bacteria. Nickel itself promotes ingrowth of those organisms being a part of several microbial metalloenzymes [12,13].

Other steels, including 200, 400, and 500 series, are also used for biomedical applications due to their reduced chromium concentration (especially 500 series steel), although less frequently than the 316 L steel.

The mechanical properties of 316 L steel predispose it for various medical applications including: pins, rods, intramedullary nails, screws and plates, and even joint prostheses. Although 316 L steel is highly resistant to corrosion, it is susceptible to stress cracking and crevice corrosion. The first originates from access to chlorides that have been found in biological fluids. The second, from the fracture of the ultrathin, protective oxide layer that passivates the material outer surface. The susceptibility to stress corrosion cracking increases with exposure to chloride-rich, biological environments. Such exposure requires stainless steel implant removal as soon as they fulfilled their function, thus reducing the material to trauma procedure applications. In addition, crevice corrosion may occur when the implant succumbs to intermittent bending. The fracture of the oxide enables the corrosion of core material resulting in the deterioration of its mechanical properties and subsequent failure. In order to protect the implant material against crevice corrosion, two approaches were recommended: implementation of a thicker oxide passivation layer on the surface, and a careful application to avoid oxide fracture. The first approach relies on special preparation of the implant’s surface. Polishing smoothens its surface, thus reducing the contact with the outer environment; chemical preparation with nitric acid thickens the oxide layer. On the other hand, electrolytic passivation (anodizing) removes free iron particles from its surface, locally increasing the concentration of chromium and nickel, that are responsible for the resistance against corrosion [14]. The second approach requires an appropriate technology of the implant manufacturing and handling. Casting or forging form the final product into the desired shape with specific mechanical properties. Casting enables complex shapes to be produced and is a relatively simple and low-cost process. On the other hand, labour-intensive and costly forging allows for the production of an implant that is much more ductile and durable. It should be mentioned, however, that cold-working strengthens the material, but also increases brittleness. Hence, the cold-worked implants (i.e., intramedullary rods), used to stabilise shafts of a long bone, are more durable, but are not suitable for bending loads as much as cast implants. 

**Figure 1:**
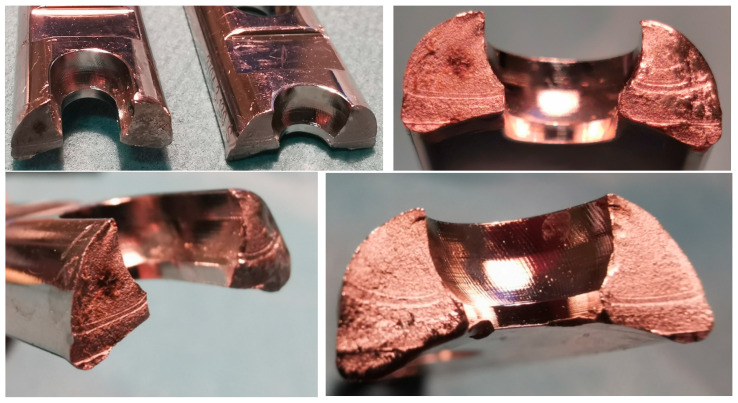
Areas of brittle and fatigue fractures at that site of the break of stainless-steel Dall-Miles Cable Plate (Stryker).

In summary, to obtain a defect free passivation layer covering an implant, several conditions should be maintained. First of all, implants should be properly designed to guarantee an appropriate stiffness, in order to withstand mechanical loading occurring in typical loading scenarios. Surgeons should perform stabilisations without tampering with the structure; that is, without the need for bending to adjust to the bone shape. It is noteworthy, that each bend of the implant, especially cyclic one, disrupts or deteriorates the passivation layer properties and leads to implant fracture (Figure 1). Additionally, special stabilisation techniques (tension band principle) defined by Pauwels in the 1930s is commonly used to reduce the risk and amplitude of plate bending during limb loads, based on the conversion of tensile forces acting over the fracture compared to compressive loads [15]. Thus, both the production and implantation technique provide the desired mechanical properties and protection against crevice corrosion that protect from unwanted electrochemical processes, resulting in loss of durability, and subsequent fracture (Figure 1).

#### 3.1.2. Titanium

Titanium and its alloys have been known since the end of the 18th century. Pure titanium, used to fabricate several alloys characterised by a relatively high hardness and corrosion resistance, found its first medical application in the 1940s as dental implants. These alloys were also used in orthopaedics due to desirable mechanical capabilities and the ability for osseointegration, defined as the capacity to bind with adjacent bone, improve implant stability and reduce the risk of losing the implant [16,17]. Additionally, a high corrosion resistance enabled the implant’s adoption for several decades without any obvious tissue irritation or toxicity effects [18]. With increased demand from aircraft factories and submarine shipyards, global production for titanium rapidly increased in the 1950s and 1960s, enabling expanded applications in the medical field. The first publication that discussed the possibility of using titanium as a surgical implant dates to 1963 [19]. The study brought an increased interest on the subject in the subsequent decade [20,21]. Low density, high strength, and high corrosion resistance predispose this metal to the production of surgical implants, especially in its beta allotropic form, and alloyed with molybdenum, vanadium, niobium, tantalum, and zirconium. Nowadays, titanium alloys have been widely used to fabricate trauma and orthopaedic implants [22,23] due to increased biocompatibility, lack of toxicity, osseointegration, high tensile strength to density ratio, and corrosion resistance. The most popular titanium alloy used for implants is aluminium-vanadium doped alloy (Ti6Al4V). Currently, practically every type of orthopaedic implant has a titanium ‘variant’, including screws, plates, intramedullary nails and rods, external fixators, and joint prostheses. Since titanium is non dielectric and does not increase in temperature when exposed to alternating magnetic fields, it is ideal as an implant as it also does not interfere with magnetic resonance imaging [24]. This significant advantage of titanium has dominated the materials’ application in traumatology and joint replacements, and practically monopolised the market of implants used in spine surgery [25,26]. Additionally, its elasticity is much more comparable to the viable bone rather than that of the steel. The similar properties of implant and bone enable to avoid non-desired strain components and an overload at the bone-implant interface, thus reducing the risk of loss or periprosthetic fracture [27]. 

To date, there is minimal evidence to suggest immune adverse reactions from titanium implants, although the possibility to activate discrete cellular reactions has been postulated, as activation of leukocyte emigration and their concentration at tissues adjacent to titanium implants have been observed in the literature [28]. An interesting finding is that leukocyte emigrations were not as severe around stainless-steel implants, possibly due to their high nickel content [29]. 

Titanium is rarely used in its pure form [30]. Nevertheless, it still serves for an implant’s coating with spongy, three-dimensional, plasma-sprayed layers, that provide at least some titanium characteristics to other materials [31]. In the vast majority of cases, the Ti6Al4V and its derivatives are used in orthopaedics. However, newly designed alloys, including TiNbZrTaSiFe [32], TiMoFe [33], and TiMoNbZr [34], are characterised by the modified or improved mechanical properties and have become an alternative to traditionally used alloys. The new generation titanium alloys exhibit greater elasticity (e.g., the Young’s modulus ca 50–65 GPa) that is similar to that of bone, which predispose them as a more suitable material for orthopaedic purposes. To manufacture intricate components from these new alloys, novel methods have increasingly been studied including the methodology of personalised, computer-designed, 3D implant “printing” using laser-beam sintering technology [35]; however, the enormous potential of this method has not been widely adopted to a large scale.

#### 3.1.3. Cobalt–Chromium-Molybdenum (CoCrMo) Alloys

316 L austenitic steel has been found to be susceptible to wear due to friction between working parts of an implant. Hence, wear resistant materials, including CoCr alloys, have been applied, often produced with some content of Mo and other metals including nickel, tungsten, and titanium. Specifically, the most common orthopaedic implant alloys contain between 62–68% Co, 27–30% Cr, 5–7% Mo, and <2.5% nickel, with an example alloy classification used for medical purposes being ASTM F75 CoCr alloy [36,37,38].

CoCr alloys were introduced in early 1900’s and have been characterised by good biocompatibility, high wear, and corrosion resistance, which result from high cobalt, molybdenum, and chromium content (almost twice that of steel). Moreover, these materials are simple to cast, and, thus, complex shaped implants could be produced at relatively low cost, without requiring further surface treatments compared to stainless steel. Thus far, several implants and medical instruments have been manufactured from CoCrMo alloys, including surgical blades and needles, cardiac valves, cases of pacemakers, and joint and dental prostheses. The material has exhibited excellent performance for working parts of joint implants, including heads of hip and condylar components for knee prostheses.

Vitallium, introduced in 1939, is one of the most popular CoCrMo alloys (65%, 30%, and 5% wt., respectively) used for the manufacture of joint replacements, starting from Charnley’s hip prosthesis [36]. It was found to be extremely durable, with orthopaedic implants manufactured from this material being in continuous use for as long as 70 years [37]. Unfortunately, the implants are susceptible to breaking during bending, showing their limited usefulness in long bone fracture stabilisations. Another disadvantage is the relatively high chromium content jeopardising immune reactions, as the percentage of the population sensitive to this metal in modern societies has increased. Nevertheless, high wear resistance, good biocompatibility, and low cost of manufacture have made CoCr alloys very popular for orthopaedic implants in the 1960s [38], with subsequent loss of interest resulting in its replacement by titanium alloys, when the number of adverse effects was found to increase [39,40]. This accelerated, when the toxicity of wear debris produced by metal-on-metal prosthesis became well known [41]. The comparison of mechanical properties for these orthopaedic surgery alloys is presented in Table 1, where typical characteristics have been summarised.

### 3.2. Ceramic Biomaterials

Aluminium and zirconium oxides (Al_2_O_3_, ZrO_2_) and mixed oxide ceramics are used to manufacture working parts of joint prostheses components. CoCr alloys are characterised by high stiffness, scratch and corrosion resistance, and good biocompatibility. The technology of their production is relatively simple and low cost. Thus, several manufacturers offer implants of/for ceramic-on-ceramic articulation systems.

The ceramic acetabulum and prosthetic head ensure low friction and a small amount of wear debris. However, they are exposed to fragmentation, when succumbing to mechanical overload. The ceramic joints could also produce an irritating squeaking while walking [60,61].

**Figure 2:**
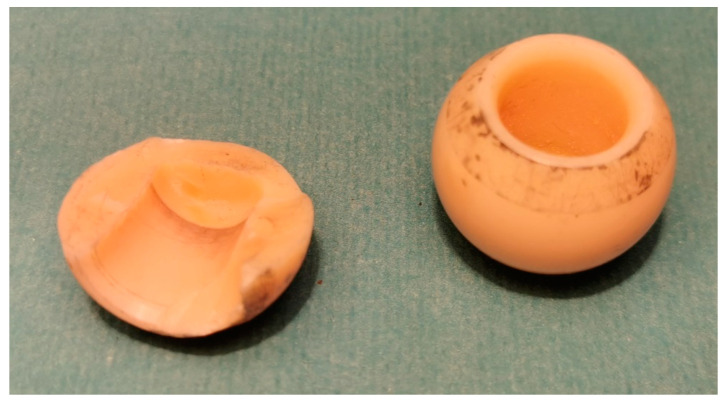
Fragmentation (break) of ceramic head of the hip prosthesis that succumbed to accidental overload with mechanical forces of high amplitude.

Primarily, alumina ceramic was the most extensively used, being replaced by zirconia due to its higher endurance and lower susceptibility to fracture. It should be highlighted, however, that all ceramics are predisposed to brittle failure when subjected to excessive mechanical loads (Figure 2). Thus, polyethylene inserts were introduced to reduce those loads. In a configuration with ultra-high-molecular-weight polyethylene (UHMWPE) acetabular insert ceramic head of the hip prosthesis exhibits reduced risk of fragmentation [61].

Recently, mixed alumina (Al_2_O_3_) and zirconia (ZrO_2_) ceramics, and those stabilised with yttrium oxide (Y_2_O_3_) or lithium silicate (Li_2_SiO_3_) were brought to the market. These ceramics are characterised by considerably higher toughness and fragmentation resistance [62,63]. Pure ZrO_2_ was found to be very brittle during the production process and cooling in particular. Thus, manufacturers alloy the material with stabilisers (calcium, magnesium, yttrium, and cerium oxides; CaO, MgO, Y_2_O_3_, and CeO_2_) which enable more durable yttria-partially stabilised tetragonal zirconia polycrystals (Y-TZP) to be obtained. Due to its biocompatibility and mechanical properties, it was found to be suitable for dental applications, although too fragile for orthopaedic implant manufacturing [64]. Thus, for orthopaedic purposes, Y-TZP is usually reinforced with Al_2_O_3_ forming alumina-toughened zirconia (AZT) that is much more resistant to cracking than Y-TZP [65]. The comparison of the mechanical and physical properties of the most popular ceramics in orthopaedics are presented in Table 2.

### 3.3. Polymeric Biomaterials

#### 3.3.1. Teflon

Tetrafluoroethylene or polytetrafluoroethylene (PTFE), better known as Teflon or Syncolone, is a synthetic fluoropolymer (C_2_F_4_)_n_ that was first manufactured in 1938. Together with its expanded form (ePTFE; Gore-Tex), it found a wide range of applications due to several unique properties [69,70,71,72,73,74]. From an orthopaedic point of view the most important are the mechanical properties. The material is non-stick and highly slippery, thus, significantly reducing the friction between working parts. Moreover, it is well tolerated in between tissue due to its extreme non-reactivity, corrosion resistance and biocompatibility [69]. Medically, Teflon was primarily used to manufacture endovascular and urinary catheters, vascular, biliary, and ocular prostheses, and as a material for soft tissue reconstructions [70]. It was used in orthopaedics to reconstruct ligamentous [71] and tendinous defects [72]. Teflon is also used in arthroplasty [73] and even to stabilise bone fractures [74]. The latter application seems to be questionable due to the inappropriate strength of the material. However, as a lubricant that reduces the friction between working parts of an implant, it seems to be an unarguably excellent alternative to other fluorine-based plastics, including ethylene tetrafluoroethylene (ETFE; brand names: Fluon, Tefzen and Texlon) [75].

#### 3.3.2. Polyethylene

Polyethylene (PE; (C_2_H_4_)_n_) is, nowadays, the most extensively used plastic in the world. It is a linear homopolymer consisting of hydrogen and carbon. It is a tough, abrasion and corrosion resistant, bioinert, self-lubricating, slippery, and semi crystalline polymer. It is also characterised by the density of 0.93 g/cm^3^, yield point of 20 MPa, and Young’s modulus of 700 GPa [76]. Polyethylene was first synthesised in 1898 by von Pechmann, while working on diazomethane [77]. Nevertheless, an attempt to synthesise it on an industrial scale was carried out in 1933 by Fawcett and Gibson. They polymerised free radicals under high temperature and pressure obtained the low-density polyethylene (LDPE). LDPE is still used for the production of plastic bags, packaging foams or plastic wraps. From the 1950s, the synthesis of the polyethylene proceeded under low pressure and temperature due to the elaboration of polymerisation catalysts. As a consequence, the high-density polyethylene (HDPE), characterised by increased hardness and tensile strength, but decreased elasticity when compared with LDPE, was obtained [78]. Neither forms were suitable for orthopaedic purposes due to their inappropriate physical properties. Thus, ultra-high molecular weight polyethylene (UHMWPE), with chains consisting of up to 200,000 monomers per molecule (HDPE only ca 1700) and molecular weight from 2 to 6 million g (HDPE: 0.05–0.25) was synthesised [79,80,81]. UHMWPE is predisposed to manufacture acetabular cups of hip and inserts of knee prostheses, as well as artificial, intervertebral discs due to good strength-to-weight ratio, low moisture absorption (almost none), extremely high impact strength, and resistance to abrasion from the high degree of polymerisation [82]. Moreover, UHMWPE is approximately 15 times more resistant to abrasion than steel and has a lower friction coefficient. Its production is also simple and cheap. It should be noted that desirable physical properties of UHMWPE, including resistance to tensile loads and shear stress, are associated with very long chains and their intermolecular attractions induced by Van der Waals forces [81]. 

UHMWPE cup with acrylic bone cement was firstly attached to the reamed space of the hip’s acetabulum by Charnley [83]. Throughout the history of UHMWPE, several attempts have been made to reinforce this polyethylene. In the 1970s, Zimmer developed polyethylene reinforced with carbon fibres. Unfortunately, it was characterised by inferior properties including reduced material strength and wear resistance in comparison to the original UHMWPE. In the late 1980s, an additional effort was made to reinforce UHMWPE by its high pressure recrystallisation (DePuy). However, the new material, called Hylamer, was of lower strength than the original UHMWPE. Its application was discontinued in the second half of the 90’s. It should be mentioned, however, that failures of Hylamer were found to be associated with radiation sterilisation [79].

**Figure 3:**
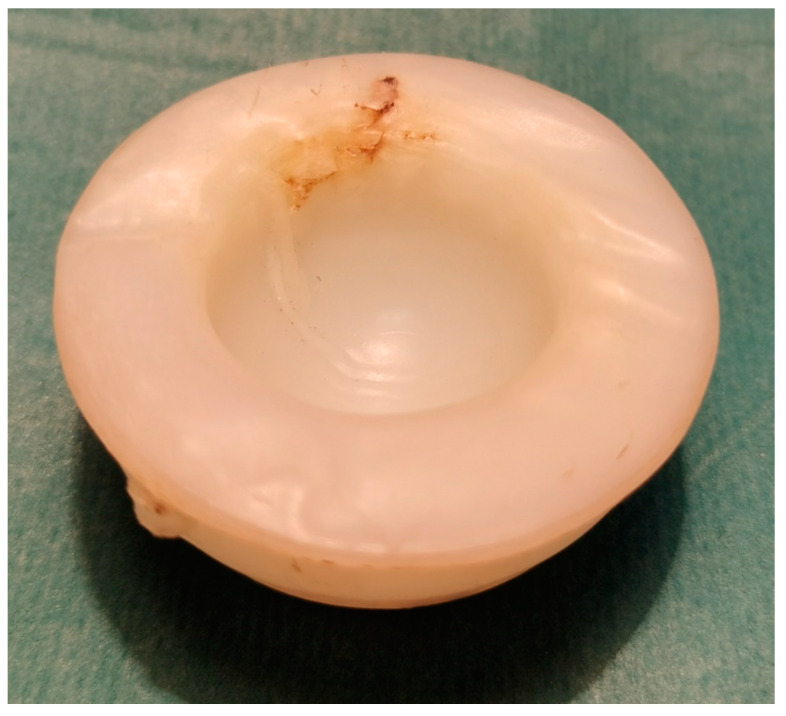
Creep and wear leading to deformation of the acetabular UHMWPE insert.

The breakthrough in polyethylene production occurred in 1998, when crosslinked UHMWPE was synthesised. Its low friction, improved mobility, reduced wear debris, and greater elasticity than most metals significantly lowered the risk of a loose implant. Polyethylene is now used in various types of joint prostheses to manufacture components working with metal and ceramic materials. Despite the fact that UHMWPE performs well as a material for moving parts of endoprostheses, its abrasive products (wear debris) activate osteoclastic bone resorption stimulating the implant’s loosing [84]. It is also exposed to creep, resulting in the implant’s deformation [85], which requires further revision procedures (Figure 3).

#### 3.3.3. Polimethylmetacrylate

Polimethylmetacrylate (PMMA; C_5_H_8_O_2_) was primarily introduced into neurosurgery and dentistry in the 1940s. For orthopaedic purposes, Judet elaborated acrylic implant reinforced with a metallic pin to restore the femoral head [86]. Charnley used the self-curing PMMA as a bone cement to anchor the prosthetic stem made from metal (1960). Currently, this material is used in orthopaedics to fix components of joint prostheses, and in the surgical treatment of osteomyelitis and infectious complications of orthopaedic implants, as well as in vertebroplasty and kyphoplasty. PMMA is also commonly used to strengthen the anchorage of implants in osteoporotic bone and to reconstruct metastatic bone defects. It is characterised by relatively low density of 1.18 g/cm^3^, ultimate tensile strength of 72 MPa, Young’s modulus of 310 GPa, and elongation of 5% [87,88]. PMMA is hard, stiff, brittle, and possess limited adhesiveness. It is usually used as a grout filling in the narrowed spaces of the bone marrow cavity in osteoporotic bone or attaching the implant to the cancellous bone filling in free spaces of its pores. When it is compressed, especially by shock forces of high amplitude, or undergoes severe bending, PMMA may break [89].

Polymerisation of PMMA proceeds as a chemical reaction between two components at room temperature. The first component, initiator, usually methylmetacrylate (MMA) or polimethylmetacrylate (PMMA) used as an amorphous powder mixed with radiopaque (e.g., barium sulfate; barite), when mixed with the second component, usually a liquid activator (MMA monomers mixed with stabiliser), begins to polymerise, forming amorphous PMMA. The process is exothermic and usually takes several minutes, where the temperature may rise up to 82.5 °C. PMMA monomers (MMA) are highly irritant and even carcinogenic even though PMMA itself is biocompatible and does not evoke tissue irritations. These materials could also lead to hypotension and lung fat embolisation. Thus, a precise amount of initial component material must be used to minimise the risk of side effects produced by unbounded monomers. 

The biomaterials for orthopaedic surgery and traumatology have a wide range of possible applications. Their advantages and limitations were presented in Table 3.

## 4. Surface Modifications

Sensitisation, toxicity, mutagenic, and teratogenic after-effects of metals correlate with the net volume of ions that have been released from the implant. Thus, manufacturers developed several processes and technologies to reduce it, including polishing (also as electro-polishing), sanding, passivation, anodisation, and covering with secondary materials. 

**Figure 4:**
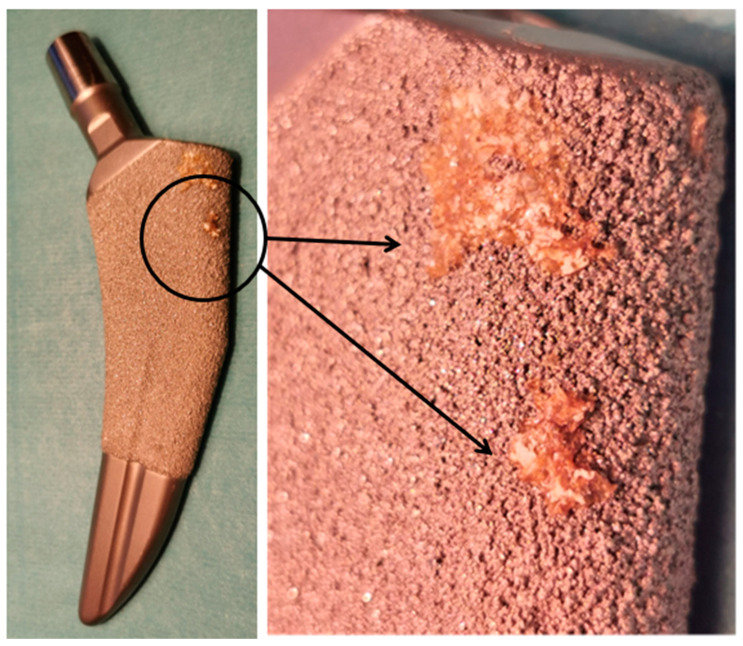
Osseointegration of the bone with implant’s titanium, porous plasma-sprayed surface (stem of the hip prosthesis; TaperLoc, Zimmer Biomet, Zug, Switzerland).

Polishing and sanding reduce the surface of the contact of an implant with an external environment, and passivation and anodisation can cover the material with an external layer protecting it from ion release. Anodising also provides a durable, highly resistant surface protecting an implant from wear off. It may also form porous structures that increase osseointegration with an adjacent bone, fixing the implant and decreasing the risk of it loosening (Figure 4) [90].

The most commonly used techniques of coating with secondary materials are plasma sprayed, hydroxyapatite and titanium nitride (TiN) ceramic coatings [91]. Moreover calcium–phosphate (CaP), carbon and diamond-like carbon coatings are under increased investigation [92,93]. The main objective of such coatings is to shield the implant from direct contact with surrounding tissue and materials, and, thus, mitigate any detrimental chemical or physical effects on the material properties and patient wellbeing. It may also be used to increase the surface porosity improving implant’s stability, and additionally, when hydroxyapatite coating is performed, also osseointegration. This technique was primarily utilised to improve dental implant stability, and soon found its use in the production of joint prostheses. Titanium-plasma spray uses titanium particles condensed and fused with the implant’s surface at high temperature produced by an electric arc. Hydroxyapatite coatings by a thermal spray process enables to implant’s surface to be covered with calcium phosphate coating of crystalline hydroxyapatite. This particular coating osseointegrates with an adjacent bone being remodelled with bone trabecula’s [94,95].

Titanium nitride (TiN) coating smoothens, hardens, and enhances abrasion and corrosion resistance of the implant’s surface decreasing its wear and deformation, when loaded. It shares properties with deeper situated materials enabling lowering cost of manufacture. Additionally, it protects the implant from contact with adjacent tissue and materials reducing exchange of metal ions. Coating of the implant with TiN reduces successfully unwanted side effects produced by chromium release from CoCrMo alloys [96]. 

Carbon coating prevents unwanted side effects of ions released from the implant, but its limited endurance reduces the number of possible orthopaedic applications [97]. Diamond-like carbon and graphene are much more promising due to their unique capabilities. They are tested to be used not only as covering materials shielding an implant from the outer environment, but also as mechanical protection and as antimicrobial drug-delivery systems [98,99,100]. Several attempts have been made to expand the use of other materials in the manufacture of orthopaedic implants, including polyethylene. Polyethylene is valued due to its slipperiness, abrasion, and corrosion resistance. However, the material hardness is not comparable with metallic components, thus limiting its deformation resistance and increased susceptibility to wear. As a consequence, several attempts have been made to improve the mechanical resistance, including modifications to the chemical structure (increased molecular weight and cross-linking) and, more recently, with reinforcement [101] and coating with more resistant materials [102].

From the early beginning of implant manufacture, the materials used in orthopaedic procedures were mainly found in industrial applications. Nevertheless, complications arising from the lack of biocompatibility were soon made apparent, and, therefore, requiring the modification of the alloys, or the development of new materials that would mitigate the issues with standardised materials. As a result, new materials, including stainless “surgical” steel, CoCrMo, and titanium alloys, were introduced to further allow the manufacture of implants characterised by much more suitable properties for medicine, thus giving much better results for treatment. Nevertheless, those materials also exhibited several disadvantages, proving the necessity to their further modification. Ideally, an appropriate material would exhibit mechanical characteristics that closely resemble the human bone and whose properties would enable the adoption of novel manufacturing techniques such as laser-beam sintering to provide the possibility of three-dimensional, personalised implants according to the patient’s stature and special requirements. Moreover, materials that could also serve as a carrier of molecular substances supporting and regulating biological processes at the implant-bone interface, including fracture healing, implant’s osseointegration, and regulation of the microbial growth, would highly advance the orthopaedics field. 

A very promising method for implant manufacturing is the use of a thin coating layer covering the outer surface of the implant produced with less sophisticated, easier to machine, and lower cost material. The possibility to modify and control surface properties at the micro and even nano-level constitute one of the major breakthroughs, and can open a new range of strategies for achieving the desired interaction with the biological environment. Another promising research direction includes the development of biologically active, absorbable polymers, and composites, that could serve as substrates or scaffolds for biologically active substances that would be able to stimulate cellular processes, namely, adhesion, activations, proliferation, and differentiation into a desired cellular lineage. Osteoblasts and bony extracellular matrix, preferably enhanced by the support of angiogenesis that would support skeletal tissue regeneration, would be desirable applications. An effective cooperation between materials scientists, biologists, and orthopaedists may lead to the development of new materials characterised by more desirable, effective, and, thus, more attractive properties. 

Nowadays, it is difficult to speculate whether metals and their alloys would be soon replaced by polymers and composites. Presumably, titanium and its alloys would continue to be accepted as a material suitable for orthopaedic purposes; however, steels and CoCr alloys seem to have exhausted their capabilities due to a rising knowledge of unacceptable side effects. Surface modifications may prolong their acceptance, although the need for the development of more appropriate materials is clear.

## 5. The Limitations in Implant Manufacturing Technologies and Applications

As has been determined in Section 2, the suitability of a material for orthopaedics requires extensive investigation, elaboration, and verification of all parameters that could prove mechanically beneficial, but of high risk, to a medical setting. In the above section, some of the most effective and widely used techniques to manufacture orthopaedic implants have been discussed. 

Initial implant technology focused on the shape and adequate mechanical strength, with chemical content and microstructure being considered to be less important. However, the role of chemical composition increased in importance with the understanding of biocompatibility. The production technology; microstructure; and, resulting from these properties, durability, elasticity and stiffness, toxicity, and irritability became crucial issues during the assessment of new implant materials. This importance increased further with implants being utilised in much younger patients than in the past, with ages of approximately 30–40 years old, requiring even greater implant longevity. Younger patients are presumed to use the implant much longer than older ones, thus succumbing longer and more intensive intoxication with released metal ions. Moreover, being capable to procreate may bring the risk of teratogenic complications.

Currently, the spectra of materials available for the production of orthopaedic implants are very limited in number. Practically, every manufacturer offers implants produced with at least three alloys, steel, cobalt-chromium, and titanium; as well as at least two types of ceramic, alumina and zirconia, and polyethylene. PMMA bone cement practically closes the short list of materials that are regularly used in orthopaedics. It should be noted that even though material candidates are limited, implant manufacturers could modify the chemical composition, incorporate specific production technologies, and apply novel coatings or surface finishing on the materials to further improve performance. 

**Figure 5:**
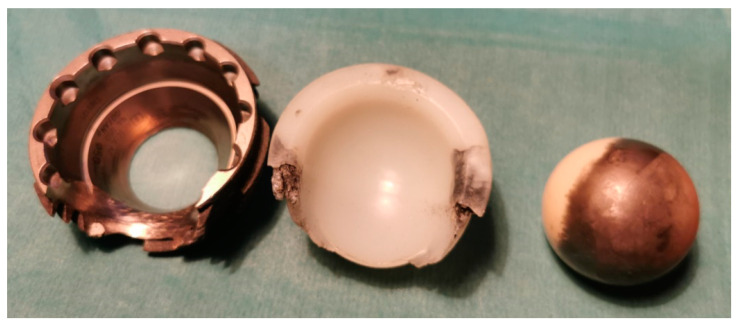

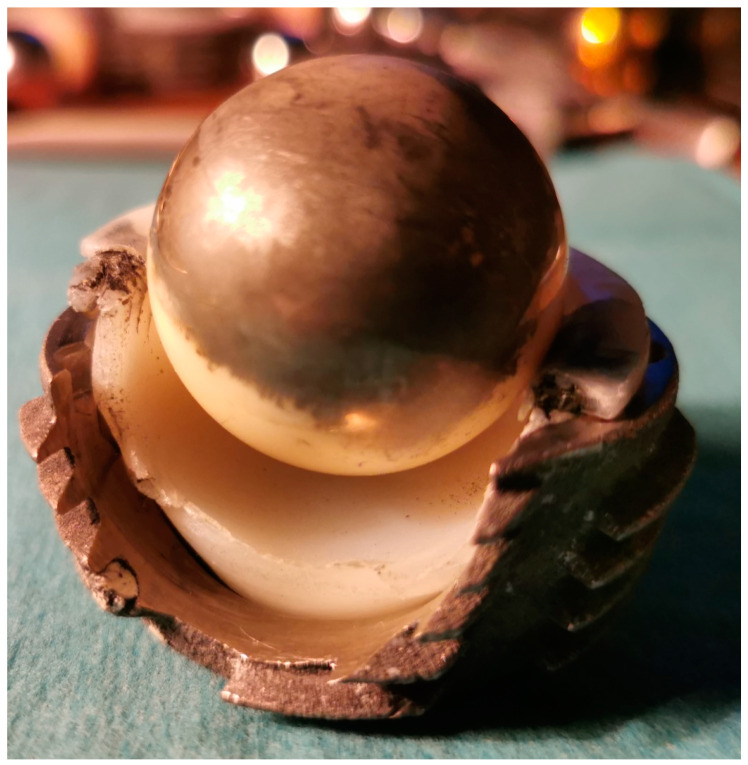
An example of the acetabular cup grinding and its UHMWPE insert by Al_2_O_3_ ceramic head during decades-long weight bearing.

**Figure 6:**
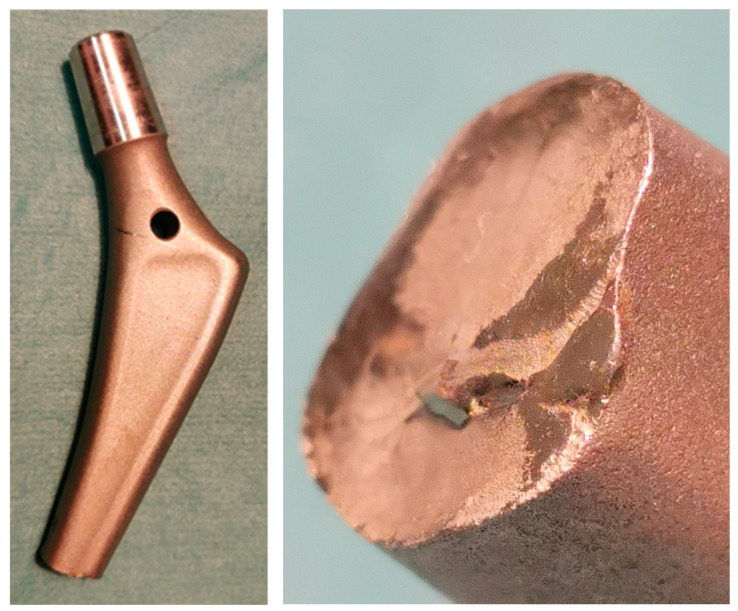
Break of the stem of the hip prosthesis and macro-photograph of the surface of its breakthrough.

**Figure 7:**
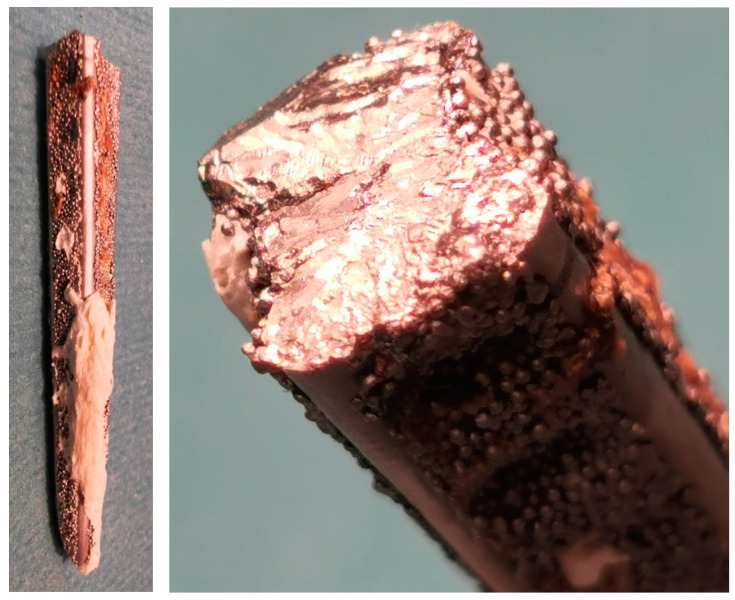
Broken stem of the hip prosthesis.

Unfortunately, even though contemporary biomaterials are suitable for the vast majority of cases, limitations exist that may compromise performance. All implants may break, exhibit dislocation or peri implant bone fracture that clearly demonstrate the challenges at the interface between a viable human bone and physical implant, commonly resulting from an unfavourable distribution of mechanical loads (Figure 5, Figure 6 and Figure 7). Figure 5 shows an example of destruction (grinding) of the acetabular cup (Munich II; Ti6Al4V Isotan-P, Aesculap) and its UHMWPE insert by Al_2_O_3_ ceramic head during decades-long weight bearing. The vast area of metallic inlay on ceramic head increases the net volume of metal ions released into surrounding tissues during this process. The range of destruction of Ti6Al4V and UHMWPE components by a macroscopically intact ceramic head proves the differences in mechanical properties between materials used to manufacture each part of the implant. On the other hand, Figure 6 presents a break of the stem of the hip prosthesis and macro-photograph of the surface of its break-through, where areas of brittle and fatigue fractures of the stem could be observed. The last example presented in Figure 7 shows a broken stem of the Mittelmeier Autophor hip prosthesis. Areas of brittle and fatigue fractures could be observed identifying mechanical overloads that destroyed the implant. One should mention that septic and aseptic loosening or the bacterial loads on the implant itself may stimulate osteolytic bone destruction. Sensitiveness, toxicity, irritancy, and even mutagenic and teratogenic capabilities corresponding with net volume of ions accumulating in-between viable tissues, as well as a conflict with magnetic resonance imaging, also exhibit further risks with implant materials. The use of implants made with metal, to which the patient is sensitive, should be avoided. The problem usually occurs when the patient is sensitive to chromium, excluding the possibility to apply implants manufactured with steel and CoCrMo alloys, or nickel (steel). In those cases, an application of implants made with titanium should be preferred. Titanium is still considered to be non-allergic, but sensitisation to this metal is also plausible, especially when multiple implantations are taken under consideration. Due to its attractive mechanical and biological properties it seems to be one of the most appropriate materials for orthopaedic purposes, and one of the safest. On the other hand, it may not pertain to recently introduced alloys that contain other metals, including niobium (Nb), tantalum (Ta) strontium (Sr), and yttrium (Y) [103], as well as antimicrobial properties of silver and copper in TiCu and TiAg coatings [104,105,106], and even newly introduced, mixed-metals, superelastic Ti16Nb3Mo1Sn and Ti19Zr10Nb1Fe alloys [107,108].

One can indicate that being sensitive to the particular metal is not equivalent to the activation of the immune reaction after its implantation. Since this reaction belongs to a type IV cell-mediated hypersensitivity, its activation is possible, when metal ions released from the implant (serving as a hapten) binds to epidermal proteins, forming conjugates that could be internalised by antigen-presenting Langerhans cells. Those cells transport antigens via lymph to the regional lymph node, where they are presented to naïve T-lymphocytes. As a consequence, proliferating and differentiating T-lymphocytes give rise to several populations of effector cells, including cytotoxic, natural killer, regulatory, helper, and memory (central, effector, tissue resident, and virtual) T-lymphocytes [109,110]. When released from the node via the lymphatic system to the circulation, they mount and regulate immune response.

Since the presentation of metal ions to Langerhans cells is crucial for the immune response, it can be mounted only when the implant contacts with skin. The reaction could also be potentially mounted by the presentation of metal ions to lung, intestine, or mucous dendritic cells, but it is practically very seldom, as orthopaedic implants are not implanted in such environments. Nevertheless, an implant’s migration may potentially be responsible for activation of the immune reaction mediated by dendritic cells. When covered with a thick layer of soft tissue or implanted deeply into the bone, e.g., as an intramedullary nail, it is unlikely that there would be an activate immune reaction. This fact explains clinically the mute courses of implantations of alloys, to whom the patient is unquestionably sensitive.

## 6. Summary and Perspectives

In summary, the characteristics of the most extensively used materials and methods used in implants have been presented and the direction of future investigations is proposed with the emphasis on novel materials to provide tailor made and highly durable bio-inert implants. Nowadays, the vast majority of implants are based on metals, due to their high durability, widespread availability, and standardised technology and knowhow for their production. Nevertheless, further improvements, such as structure or surface modification, or the reinforcement with more elastic and durable materials possess practical limitations. Polymers and composites may be found to be more suitable in the future as there many candidates that surpass metals in terms of elasticity and durability, without the risk of sensitisation and intoxication. Polyethylene is a promising material as it is bio-inert, well-tolerated in the viable tissues, and is corrosion and abrasion resistant. Moreover, the production is relatively cheap with a great number of possibilities to synthesise new polymer materials. Thus, presumably, polymers could serve as an alternative to metals and their alloys, for orthopaedic purposes in the near future.

## Figures and Tables

**Table 1:** Physical characteristics of bone, PTFE, and most the extensively used orthopaedic surgery alloys.

	Implant Type	Yield Point [MPa]	Ultimate Tensile Strength [MPa]	Young’s Modulus [GPa]	Elongation [%]	References
Bone	bone		130–205 MPa	17.9–18.2		[42,43]
Steel	316 L	170–750	465–950	205–210	30–70	[44]
Ti and alloys	CP-titanium	170–480	240–550	105	15–24	[45,46,47,48,49,50,51,52,53,54,55]
	Ti6Al4V	795–875	895–965	100–114	10	
	Ti6Al7Nb	795	860	105	10	
	Ti5Al2.5Fe	820	900	110	6	
	Ti3Al2.5V	585	690	100	15	
	Ti13Nb13Zr	836–908	937–1037	79–84	42–44	
	Ti12Mo6Zr2Fe	1000–1060	1060–1100	14–85	18–22	
	Ti24Nb4Zr8Sn	570–700	755–830	46–55	13–15	
CoCrMo alloys	Cast 28Co6CrMo	450	655	210–250	8	[56,57,58]
	Wrought Co28Cr6Mo	517–827	897–1192	220	12–20	
	Co28Cr6Mo Forging	827	1172	220–230	12	
PTFE	PTFE	4.6–7.8	7.8–11.1	42–59	20–29	[59]

**Table 2:** Physical characteristics of the most popular ceramics in orthopaedics.

Implant Type	Density [g/cm^3^]	Microhardness [HV]	Young’s Modulus [GPa]	Bending Strength [MPa]	Toughness K1C [MPa × m^1/2^]	References
Y-ZPT	6	1000–1300	200	1200	9–10	[66,67,68]
zirconia-toughened alumina (ZTA)		1460–1620	236–254	500–760	7–7.2	
alumina-toughened zirconia (AZT)	5.5	2000–2200	358–368	420–460	3.9	

**Table 3:** Advantages and disadvantages of biomaterials [12,13,14,15,16,17,18,19,20,21,22,23,24,25,26,27,28,29,30,31,32,33,34,35,36,37,38,39,40,41,42,43,44,45,46,47,48,49,50,51,52,53,54,55,56,57,58,59,60,61,62,63,64,65,66,67,68,69,70,71,72,73,74,75,76,77,78,79,80,81,82,83,84,85,86,87,88,89].

Material	Advantages	Disadvantages
Steel	High material strength Good ductility	Corrosive Aseptic loosening Inadequate wear resistance
Titanium alloys	High biocompatibility Low density Corrosion resistance	Poor tribological properties Low wear resistance Toxic effect of aluminium and vanadium
CoCrMo alloys	High material strength High wear resistance Corrosion resistance	Allergy consideration with nickel, chrome and cobalt
Ceramics	High hardness Wear resistance Good wettability Good biocompatibility	Brittle High stiffness Low flexibility
Polymers	Low density Biodegradable Easy fabrication Flexible	Hard to sterilise Poor tribological properties Absorb water and proteins

## Data Availability

All data are available upon request. The data presented in this study are available on request from the corresponding author.

## References

[1] Marin E., Boschetto F., Pezzotti G. (2020). Biomaterials and biocompatibility: An historical overview. J. Biomed. Mater. Res. Part A.

[2] Bartoníček J. (2010). Early history of operative treatment of fractures. Arch. Orthop. Trauma. Surg..

[3] Tchounwou P.B., Yedjou C.G., Patlolla A.K., Sutton D.J. (2012). Heavy metal toxicity and the environment. Mol. Clin. Environ. Toxicol..

[4] Abdalla S.S.I., Katas H., Azmi F., Busra M.F.M. (2020). Antibacterial and Anti-Biofilm Biosynthesised Silver and Gold Nanoparticles for Medical Applications: Mechanism of Action, Toxicity and Current Status. Curr. Drug Deliv..

[5] Sherman W.O. (1912). Vanadium steel plates and screws. Surg Gynecol. Obstet..

[6] Punj S., Singh J., Singh K. (2021). Ceramic biomaterials: Properties, state of the art and future prospectives. Ceram. Int..

[7] Ambrose C.G., Hartline B.E., Clanton T.O., Lowe W.R., McGarvey W.C., Puoci F. (2014). Polymers in Orthopaedic Surgery. Advanced Polymers in Medicine.

[8] Beaupré G.S., Csongradi J.J. (1996). Refracture Risk After Plate Removal in the Forearm. J. Orthop. Trauma.

[9] Fransway A.F., Zug K.A., Belsito D.V., DeLeo V.A., Fowler J.F., Maibach H.I., Marks J.G., Mathias C.T., Pratt M.D., Rietschel R.L. (2013). North American Contact Dermatitis Group Patch Test Results for 2007–2008. Dermatitis.

[10] Wu Y., Kong L. (2020). Advance on toxicity of metal nickel nanoparticles. Environ. Geochem. Health.

[11] Bradberry S.M., Wilkinson J.M., Ferner R.E. (2014). Systemic toxicity related to metal hip prostheses. Clin. Toxicol..

[12] Yatera K., Morimoto Y., Ueno S., Noguchi S., Kawaguchi T., Tanaka F., Suzuki H., Higashi T. (2018). Cancer Risks of Hexavalent Chromium in the Respiratory Tract. J. UOEH.

[13] Kaluarachchi H., Chung K.C.C., Zamble D.B. (2010). Microbial nickel proteins. Nat. Prod. Rep..

[14] Lautenschlager E.P., Sarker N.K., Acharya A., Galante J.O., Rostoker W. (1974). Anodic polarization of porous fiber metals. J. Biomed. Mater. Res..

[15] Pauwels F. (1966). Überraschende Erfolge durch die Anwendung einer Zuggurtung bei der Patellarfraktur. Langenbeck’s Arch. Chir..

[16] Levanthal G.C. (1951). Titanium: A metal for surgery. J. Bone Jt. Surg..

[17] Wang H., Su K., Su L., Liang P., Ji P., Wang C. (2019). Comparison of 3D-printed porous tantalum and titanium scaffolds on osteointegration and osteogenesis. Mater. Sci. Eng. C.

[18] Neuman G., Spångberg L., Langeland S. (1975). Methodology and criteria in the evaluation of dental implants. J. Endod..

[19] Golberg S.Z., Vainer E.l., Vasiljeva G.S. (1963). Study of the possibility of using titanium in medical technology. Med. Prom. SSSR.

[20] Galante J., Rostoker W. (1972). Corrosion-related Failures in Metallic Implants. Clin. Orthop. Relat. Res..

[21] Williams D.F. (1972). Corrosion and corrosion prevention in orthopaedic implants. Proc. R. Soc. Med..

[22] Woo S.L.-Y., Lothringer K.S., Akeson W.H., Coutts R.D., Woo Y.K., Simon B.R., Gomez M.A. (1983). Less rigid internal fixation plates: Historical perspectives and new concepts. J. Orthop. Res..

[23] Semlitsch M. (1982). Concept and material properties of a cementless hip prosthesis system with Al2O3 ceramic ball heads and wrought Ti-6Al-4V Stems. Arch. Orthop. Trauma. Surg..

[24] Henk C.B., Brodner W., Grampp S., Breitenseher M., Thurnher M., Mostbeck G.H., Imhof H. (1999). The Postoperative Spine. Top. Magn. Reson. Imaging.

[25] Li Q., Min X., Bai P., Wang W., Tao X., Zhong G., Bai S., Zhao J. (2018). Microstructure, mechanical properties and springback behaviour of Ti-6Al-4V alloy connection rod for spinal fixation device. Mater. Sci. Eng. C.

[26] García-Gareta E., Hua J., Orera A., Kohli N., Knowles J.C., Blunn G.W. (2017). Biomimetic surface functionalization of clinically relevant metals used as orthopaedic and dental implants. Biomed. Mater..

[27] Head W.C., Bauk D.J., Emerson R.H. (1995). Titanium as the material of choice for cementless femoral components in total hip arthroplasty. Clin. Orthop. Relat. Res..

[28] Radley G., Pieper I.L., Thomas B., Hawkins K., Thornton C.A. (2018). Artificial shear stress effects on leukocytes at a biomaterial interface. Artif. Organs.

[29] Perren S.M., Regazzoni P., Fernandez A.A. (2017). How to Choose between the Implant Materials Steel and Titanium in Orthopaedic Trauma Surgery: Part 2—Biological Aspects. Acta Chir. Orthop. Traumatol. Cech..

[30] Shah F.A., Trobos M., Thomsen P., Palmquist A. (2016). Commercially pure titanium (cp-Ti) versus titanium alloy (Ti6Al4V) materials as bone anchored implants—Is one truly better than the other?. Mater. Sci. Eng. C.

[31] Wypych A., Siwak P., Andrzejewski D., Jakubowicz J. (2018). Titanium Plasma-Sprayed Coatings on Polymers for Hard Tissue Applications. Materials.

[32] Kopova I., Stráský J., Harcuba P., Landa M., Janeček M., Bačákova L. (2016). Newly developed Ti–Nb–Zr–Ta–Si–Fe biomedical beta titanium alloys with increased strength and enhanced biocompatibility. Mater. Sci. Eng. C.

[33] Abdelrhman Y., Gepreel M.A.-H., Kobayashi S., Okano S., Okamoto T. (2019). Biocompatibility of new low-cost (α + β)-type Ti-Mo-Fe alloys for long-term implantation. Mater. Sci. Eng. C.

[34] Nnamchi P., Obayi C., Todd I., Rainforth M. (2016). Mechanical and electrochemical characterisation of new Ti–Mo–Nb–Zr alloys for biomedical applications. J. Mech. Behav. Biomed. Mater..

[35] Ghosh S., Abanteriba S., Wong S., Houshyar S. (2018). Selective laser melted titanium alloys for hip implant applications: Surface modification with new method of polymer grafting. J. Mech. Behav. Biomed. Mater..

[36] Charnley J. (1961). ARTHROPLASTY OF THE HIP A New Operation. Lancet.

[37] https://www.guinnessworldrecords.com/news/2019/11/the-inspiring-story-of-the-man-whose-pioneering-hip-replacement-has-lasted-a-reco-597819.

[38] Bardos D.I., Kossowsky R., Kossowsky N. (1986). Metallurgy of orthopaedic implants. Materials Sciences and Implant Orthopaedic Surgery.

[39] Gaillard M.D., Gross T.P. (2017). Metal-on-metal hip resurfacing in patients younger than 50 years: A retrospective analysis. J. Orthop. Surg. Res..

[40] Munemoto M., Grammatopoulos G., Tanaka Y., Gibbons M., Athanasou N.A. (2017). The pathology of failed McKee-Farrar implants: Correlation with modern metal-on-metal-implant failure. J. Mater. Sci. Mater. Electron..

[41] Ho J.H., Leikin J.B., Dargan P.I., Archer J.R.H., Wood D.M., Brent J. (2017). Metal-on-Metal Hip Joint Prostheses: A Retrospective Case Series Investigating the Association of Systemic Toxicity with Serum Cobalt and Chromium Concentrations. J. Med. Toxicol..

[42] Morgan E.F., Unnikrisnan G.U., Hussein A.I. (2018). Bone Mechanical Properties in Healthy and Diseased States. Annu. Rev. Biomed. Eng..

[43] Lakes R. Elastic Anisotropy of Bone. http://silver.neep.wisc.edu/~lakes/BoneAniso.html.

[44] Navarro M., Michiardi A., Castaño O., Planell J.A. (2008). Biomaterials in orthopaedics. J. R. Soc. Interface.

[45] Zhang L.-C., Chen L.-Y. (2019). A Review on Biomedical Titanium Alloys: Recent Progress and Prospect. Adv. Eng. Mater..

[46] Zhang L., Klemm D., Eckert J., Hao Y., Sercombe T. (2011). Manufacture by selective laser melting and mechanical behavior of a biomedical Ti–24Nb–4Zr–8Sn alloy. Scr. Mater..

[47] Niinomi M. (1998). Mechanical properties of biomedical titanium alloys. Mater. Sci. Eng. A.

[48] Davis J. (2003). Handbook of Materials for Medical Devices.

[49] Chen Q., Thouas G.A. (2015). Metallic implant biomaterials. Mater. Sci. Eng. R Rep..

[50] Biesiekierski A., Lin J., Li Y., Ping D., Yamabe-Mitarai Y., Wen C. (2016). Investigations into Ti–(Nb,Ta)–Fe alloys for biomedical applications. Acta Biomater..

[51] Santos P.F., Niinomi M., Liu H., Cho K., Nakai M., Trenggono A., Champagne S., Hermawan H., Narushima T. (2016). Improvement of microstructure, mechanical and corrosion properties of biomedical Ti-Mn alloys by Mo addition. Mater. Des..

[52] Gepreel M.A.-H. (2015). Improved Elasticity of New Ti-Alloys for Biomedical Applications. Mater. Today Proc..

[53] Kusano Y., Inamura T., Kanetaka H., Miyazaki S., Hosoda H. (2010). Phase Constitution and Mechanical Properties of Ti-(Cr, Mn)-Sn Biomedical Alloys. Mater. Sci. Forum.

[54] Talling R., Dashwood R., Jackson M., Dye D. (2009). Compositional variability in gum metal. Scr. Mater..

[55] Ijaz M.F., Héraud L., Castany P., Thibon I., Gloriant T. (2020). Superelastic Behavior of Biomedical Metallic Alloys. Met. Mater. Trans. A.

[56] (2018). Standard Specification for Cobalt-28 Chromium-6 Molybdenum Alloy Castings and Casting Alloy for Surgical Implants (UNS R30075).

[57] (2020). Standard Specification for Wrought Cobalt-28Chromium-6Molybdenum Alloys for Surgical Implants (UNS R31537, UNS R31538, and UNS R31539).

[58] (2019). Standard Specification for Cobalt-28 Chromium-6 Molybdenum Alloy Forgings for Surgical Implants (UNS R31537, R31538, R31539).

[59] Catanese J., Cooke D., Maas C., Pruitt L. (2002). Mechanical properties of medical grade expanded polytetrafluoroethylene: The effects of internodal distance, density, and displacement rate. J. Biomed. Mater. Res..

[60] Kocadal O., Ozler T., Bolukbasi A.E.T., Altintas F. (2019). Non-traumatic Ceramic Head Fracture in Total Hip Arthroplasty with Ceramic-on-Ceramic Articulation at Postoperative 16th Years. Hip Pelvis.

[61] Sentuerk U., Von Roth P., Perka C., Surgeon O. (2016). Ceramic on ceramic arthroplasty of the hip. Bone Jt. J..

[62] Zhang Y., Lawn B. (2017). Novel Zirconia Materials in Dentistry. J. Dent. Res..

[63] D’Addazio G., Santilli M., Rollo M.L., Cardelli P., Rexhepi I., Murmura G., Husain N.A.-H., Sinjari B., Traini T., Özcan M. (2020). Fracture Resistance of Zirconia-Reinforced Lithium Silicate Ceramic Crowns Cemented with Conventional or Adhesive Systems: An In Vitro Study. Materials.

[64] Mussano F., Genova T., Munaron L., Faga M.G., Carossa S., Jawad M.A., Almasri A. (2016). Ceramic Biomaterials for Dental Implants: Current Use and Future Perspectives. Dental Implantology and Biomaterial.

[65] De Freitas B.X., Alves M.F.R.P., Santos C., Ramos A.S., Ramos E.C.T., Strecker K. (2020). Mechanical properties of biocompatible Y-TZP/Al_2_O_3_ composites obtained from mechanically alloyed powders. J. Braz. Soc. Mech. Sci. Eng..

[66] Duraccio D., Mussano F., Faga M.G. (2015). Biomaterials for dental implants: Current and future trends. J. Mater. Sci..

[67] Faga M., Vallée A., Bellosi A., Mazzocchi M., Thinh N.N., Martra G., Coluccia S. (2012). Chemical treatment on alumina–zirconia composites inducing apatite formation with maintained mechanical properties. J. Eur. Ceram. Soc..

[68] Spies B.C., Sauter C., Wolkewitz M., Kohal R.-J. (2015). Alumina reinforced zirconia implants: Effects of cyclic loading and abutment modification on fracture resistance. Dent. Mater..

[69] Harrison J.H., Swanson D.S., Lincoln A.F. (1957). A Comparison of the Tissue Reactions to Plastic Materials. AMA Arch. Surg..

[70] Ludington L.G., Woodward E.R. (1959). Use of teflon mesh in the repair of musculofascial defects. Surgery.

[71] Butler H.C. (1964). Teflon as a prosthetic ligament in repair of ruptured anterior cruciate ligaments. Am. J. Vet. Res..

[72] Williams R.D., August S.F. (1964). Experimental evaluation of a Teflon tendon prosthesis. Am. J. Surg..

[73] Charnley J. (1966). Using Teflon in arthroplasty of the hip-joint. J. Bone Jt. Surg. Am..

[74] Trubnikov V.F. (1963). Primenenie plastmassy tetraftoretilena (ftoroplast-4) s tseliu osteosinteza. Ortop. Travmatol. Protez..

[75] Burns K.M. (1963). The “Fluon” arthroplasty: Treatment note. Physiotherapy.

[76] Buechel F.F., Pappas M.J. (2015). Properties of Materials Used in Orthopaedic Implant Systems. Principles of Human Joint Replacement.

[77] Von Pechmann H. (1898). Über Diazomethan und Nitrosoacylamine. Berichte der Deutschen Chemischen Gesellschaft zu Berlin.

[78] Kalia S., Avérous L. (2011). Biopolymers: Biomedical and Environmental Applications.

[79] Ruso J.M., Messina P.V. (2021). Biopolymers for Medical Applications.

[80] Li S., Burstein A.H. (1994). Ultra-high molecular weight polyethylene. The material and its use in total joint implants. J. Bone Jt. Surg..

[81] Kurtz S.M. (2004). The UHMWPE Handbook: Ultra-High Molecular Weight Polyethylene in Total Joint Replacement.

[82] Veruva S.Y., Lanman T.H., Isaza J.E., MacDonald D.W., Kurtz S.M., Steinbeck M.J. (2015). UHMWPE Wear Debris and Tissue Reactions Are Reduced for Contemporary Designs of Lumbar Total Disc Replacements. Clin. Orthop. Relat. Res..

[83] Dowling J., Atkinson J.R., Dowson D., Charnley J. (1978). The characteristics of acetabular cups worn in the human body. J. Bone Jt. Sur..

[84] Geerdink C.H., Grimm B., Vencken W., Heyligers I.C., Tonino A.J. (2008). Cross-linked Compared with Historical Polyethylene in THA: An 8-year Clinical Study. Clin. Orthop. Relat. Res..

[85] Deng M., Latour R.A., Ogale A.A., Shalaby S.W. (1998). Study of creep behavior of ultra-high-molecular-weight polyethylene systems. J. Biomed. Mater. Res..

[86] Judet R. (1951). Hip Reconstruction by Acrylic Prosthesis. BMJ.

[87] Ali U., Karim K.J.B.A., Buang N.A. (2015). A Review of the Properties and Applications of Poly (Methyl Methacrylate) (PMMA). Polym. Rev..

[88] Kutz M. (2002). Handbook of Materials Selection.

[89] Frazer R.Q., Byron R.T., Osborne P.B., West K.P. (2005). PMMA: An Essential Material in Medicine and Dentistry. J. Long-term Eff. Med Implant..

[90] İzmir M., Ercan B. (2019). Anodization of titanium alloys for orthopedic applications. Front. Chem. Sci. Eng..

[91] Łapaj Ł., Rozwalka J. (2019). Retrieval analysis of TiN (titanium nitride) coated knee replacements: Coating wear and degradation in vivo. J. Biomed. Mater. Res. Part B Appl. Biomater..

[92] Zhang X., Lv Y., Fu S., Wu Y., Lu X., Yang L., Liu H., Dong Z. (2020). Synthesis, microstructure, anti-corrosion property and biological performances of Mn-incorporated Ca-P/TiO2 composite coating fabricated via micro-arc oxidation. Mater. Sci. Eng. C.

[93] Milan P.B., Khamseh S., Zarintaj P., Ramezanzadeh B., Badawi M., Morisset S., Vahabi H., Saeb M.R., Mozafari M. (2020). Copper-enriched diamond-like carbon coatings promote regeneration at the bone–implant interface. Heliyon.

[94] Aviles T., Hsu S.-M., Clark A., Ren F., Fares C., Carey P.H., Esquivel-Upshaw J.F. (2020). Hydroxyapatite Formation on Coated Titanium Implants Submerged in Simulated Body Fluid. Materials.

[95] Ong J., Chan D.C.N. (2017). A Review of Hydroxapatite and its use as a Coating in Dental Implants. Crit. Rev. Biomed. Eng..

[96] Van Hove R.P., Sierevelt I.N., van Royen B.J., Nolte P.A. (2015). Titanium-Nitride Coating of Orthopaedic Implants: A Review of the Literature. BioMed Res. Int..

[97] Zielinski J., Lacy T.A., Phillips J.H. (2014). Carbon Coated Implants as a New Solution for Metal Allergy in Early-Onset Scoliosis: A Case Report and Review of the Literature. Spine Deform..

[98] Zhang M., Xie T., Qian X., Zhu Y., Liu X. (2020). Mechanical Properties and Biocompatibility of Ti-doped Diamond-like Carbon Films. ACS Omega.

[99] Döring J., Crackau M., Nestler C., Welzel F., Bertrand J., Lohmann C.H. (2019). Characteristics of different cathodic arc deposition coatings on CoCrMo for biomedical applications. J. Mech. Behav. Biomed. Mater..

[100] Gallo J., Holinka M., Moucha C.S. (2014). Antibacterial Surface Treatment for Orthopaedic Implants. Int. J. Mol. Sci..

[101] Bhusari S.A., Sharma V., Bose S., Basu B. (2019). HDPE/UHMWPE hybrid nanocomposites with surface functionalized graphene oxide towards improved strength and cytocompatibility. J. R. Soc. Interface.

[102] Harrasser N., Jüssen S., Obermeier A., Kmeth R., Stritzker B., Gollwitzer H., Burgkart R. (2016). Antibacterial potency of different deposition methods of silver and copper containing diamond-like carbon coated polyethylene. Biomater. Res..

[103] https://www.daido.co.jp/en/products/titanium/medical/index.html.

[104] Wang X., Dong H., Liu J., Qin G., Chen D., Zhang E. (2019). In vivo antibacterial property of Ti-Cu sintered alloy implant. Mater. Sci. Eng. C.

[105] Shi A., Zhu C., Fu S., Wang R., Qin G., Chen D., Zhang E. (2019). What controls the antibacterial activity of Ti-Ag alloy, Ag ion or Ti2Ag particles?. Mater. Sci. Eng. C.

[106] Alt V. (2017). Antimicrobial coated implants in trauma and orthopaedics–A clinical review and risk-benefit analysis. Injury.

[107] Al-Zain Y., Yamamoto A., AlAjlouni J.M., Al-Abbadi M.A., Al-Sayyed M.R., Aloweidi A.S., Kim H.Y., Miyazaki S. (2019). Corrosion behavior, in vitro and in vivo biocompatibility of a newly developed Ti–16Nb–3Mo–1Sn superelastic alloy. Mater. Sci. Eng. C.

[108] Xue P., Li Y., Li K., Zhang D., Zhou C. (2015). Superelasticity, corrosion resistance and biocompatibility of the Ti–19Zr–10Nb–1Fe alloy. Mater. Sci. Eng. C.

[109] Nguyen Q.P., Deng T.Z., Witherden D.A., Goldrath A.W. (2019). Origins of CD4^+^ circulating and tissue-resident memory T-cells. Immunology.

[110] Shin H., Iwasaki A. (2013). Tissue-resident memory T cells. Immunol. Rev..

